# Association Between Factors Involved in Bone Remodeling (Osteoactivin and OPG) With Plasma Levels of Irisin and Meteorin-Like Protein in People With T2D and Obesity

**DOI:** 10.3389/fendo.2021.752892

**Published:** 2021-10-29

**Authors:** Preethi Cherian, Irina Al-Khairi, Mohammad Jamal, Suleiman Al-Sabah, Hamad Ali, Carol Dsouza, Eman Alshawaf, Waleed Al-Ali, Ghanim Al-Khaledi, Fahd Al-Mulla, Mohamed Abu-Farha, Jehad Abubaker

**Affiliations:** ^1^ Department of Biochemistry and Molecular Biology, Dasman Diabetes Institute, Kuwait City, Kuwait; ^2^ Department of Surgery, Faculty of Medicine, Health Sciences Centre, Kuwait University, Sulaibekhat, Kuwait; ^3^ Department of Pharmacology & Toxicology, Faculty of Medicine, Kuwait University, Safat, Kuwait; ^4^ Department of Genetic and Bioinformatics, Dasman Diabetes Institute, Kuwait City, Kuwait

**Keywords:** osteoactivin, OPG, irisin, meteorin-like protein, SPARC, syndecan-4, obesity, type-2 diabetes

## Abstract

The musculoskeletal system consisting of bones and muscles have been recognized as endocrine organs secreting hormones that are involved in regulating metabolic and inflammatory pathways. Obesity and type 2 diabetes (T2D) are associated with several musculoskeletal system complications. We hypothesized that an interaction exists between adipomyokines namely, irisin and METRNL, and various molecules involved in bone remodeling in individuals with obesity and T2D. A total of 228 individuals were enrolled in this study, including 124 non-diabetic (ND) and 104 T2D. A Multiplex assay was used to assess the level of various osteogenic molecules namely osteoactivin, Syndecan, osteoprotegerin (OPG) and osteonectin/SPARC. Our data shows elevated levels of Osteoactivin, Syndecan, OPG and SPARC in T2D as compared to ND individuals (p ≤ 0.05). Using Spearman’s correlation, a positive correlation was observed between irisin and Osteoactivin as well as OPG (p < 0.05). Similarly, a positive association was observed between METRNL and Osteoactivin (p < 0.05). The strong positive association shown in this study between irisin, METRNL and various molecules with osteogenic properties emphasize a possible interaction between these organs. This report suggests that having a dysregulation in the level of the aforementioned molecules could potentially affect the development of bone and muscle related complications that are associated with obesity and T2D.

## Introduction

The musculoskeletal system consists mainly of bones and muscles that interacts physically and mechanically to support the body and allow its movement in addition to providing protection to vital organs. More recently, both bones and muscles have been recognized as endocrine organs secreting hormones that are involved in regulating metabolic and inflammatory pathways (1). As a modern epidemic, diabesity is associated with metabolic dysregulation and chronic inflammation (2). Furthermore, obesity and diabetes cause several complications associated with the musculoskeletal system including osteoporosis and sarcopenia (3, 4). Sarcopenia is defined as the progressive loss of muscle mass that is also associated with a decline in muscle function. Hallmarks of diabetes and metabolic diseases including insulin resistance, oxidative stress as well as lipid infiltration in muscles amongst the pathophysiological causes of sarcopenia (4). On the other hand, osteoporosis is a bone disorder that occurs when there is a decrease in bone density, increasing the risk of fracture. Diabetes has been strongly associated with an increased risk of osteoporosis-associated fractures (5). Similarly, obesity is closely interconnected with bone metabolism (6). Several factors affecting muscle mass and various osteogenic molecules have been shown to be dysregulated with diabetes and obesity (7).

Adipomyokines have recently been identified as molecules that are secreted by the skeletal muscle and adipose tissue and play an important role in modulating metabolic pathways (8–10). These molecules are released in response to several stimuli, including muscle contraction during physical activity and/or nutritional changes (11, 12). They have also been shown to mediate muscle growth (myogenesis) and regeneration. These molecules are part of a complex network that mediates crosstalk among muscles, liver, adipose tissue, brain and bone and they have also been shown to be involved in promoting beige fat thermogenesis (13). Our group previously reported an increase in the levels of two adipomyokines, irisin and Meteorin-like (METRNL) in T2D, which was further exacerbated with obesity (14, 15).

Irisin and METRNL are two adipomyokines that have recently gained interest because of their role in increasing thermogenesis following exercise and cold exposure (16, 17). Studies have also shown that irisin plays an important role in modulating bone metabolism through exercise (16). Furthermore, Colaianni et al. reported that irisin targets bone cells directly and enhances the differentiation of bone marrow stromal cells into mature osteoblasts (18). In a follow up study by the same group, it was demonstrated that injecting recombinant irisin (r-irisin) into mice lead to increased cortical bone mass and strength. This was attributed to a direct effect of irisin on osteoblastic bone formation, occurring mainly through suppressing a Wnt signaling inhibitor such as sclerostin (Sost) (19). The beneficial effects of irisin were further shown by Zhang et al. where the induction of osteoblastogenesis and inhibition of osteoclastogenesis was achieved by an intraperitoneal (IP) administration of recombinant irisin in bone cell lines. They reported that a threefold increase of irisin levels in circulation for 2 weeks reiterates the anabolic effects of exercise in the murine skeletal system (20). Moreover, circulating serum level of irisin has been shown to be positively correlated with bone mineral status (21). The adipomyokine METRNL was shown to have a unique expression pattern in bone and the anomalous expression of METRNL was implicated to play an inhibitory role in bone cell differentiation (22).

Considering the involvement of adipomyokines in bone metabolism and the association between diabesity and bone loss, the aim of this study was to investigate the potential link between irisin, METRNL and various molecules involved in bone remodeling in people with obesity and T2D.

## Materials and Methods

### Study Population

This study included 228 adult men and women with and without T2D that were further classified according to their body weight: 104 people with T2D of which, 38 were non-obese (Nob) and 66 individuals were obese (ob), while the non-diabetic (ND) population consisted of 124 individuals (73 Nob and 51 ob). Diabetes was defined as having fasting blood glucose (FBG) ≥ 7 mmol/L, receiving anti-diabetes treatment, or self-reported T2D (14, 23). Body mass index (BMI) score was used to indicate the presence or absence of obesity with BMI > 30 kg/m^2^ indicating people with obesity and a BMI score between 20 and 30 kg/m^2^ indicating the absence of obesity. A written informed consent was obtained from all study participants. The study excluded people with morbid obesity (BMI > 40 kg/m^2^) who had chronic conditions and/or individuals who were placed on medication/supplements known to influence body composition or bone mass. The study was approved by the ethical review board of Dasman Diabetes Institute and was conducted in accordance with the ethical guidelines of the Declaration of Helsinki (Project#: RA-2016-045).

### Blood Sampling

Venous blood samples were collected using Vacutainer EDTA tubes after fasting for a minimum of 8h and blood was collected in the morning. Collected blood was centrifuged at 400 × g for 10 min at room temperature and plasma was separated, aliquoted, and stored at −80°C until the assay was performed.

### Measuring Levels of METRNL and Irisin in Plasma

Plasma levels of METRNL and irisin were measured using the Enzyme Linked Immunosorbent Assay (ELISA). Plasma samples were thawed on ice and centrifuged for 5 min at 10,000 ×g at 4°C to remove any remaining cells or platelets. Plasma level of METRNL was measured using the Human METRNL ELISA kit (LifeSpan BioSciences, Inc., Seattle, WA, USA. Cat# LS-F13315). The samples were diluted 10× with sample diluent. ELISA was performed according to the manufacturer’s instructions. The intra-assay coefficient of variation was 5.0%–10.0% while the inter-assay coefficient of variation was < 10%. Plasma level of irisin was measured using the irisin recombinant ELISA kit (Phoenix Pharmaceuticals, Inc., Burlingame, CA, USA. Cat #EK-067-29) following the manufacturer’s kit instructions. Plasma samples were diluted 40× with the 1× assay buffer (provided in the kit). The intra-assay coefficient for this ELISA assay was 1.0%–7.0%, while the inter-assay coefficient was < 20%.

### Measuring the Levels of Osteogenic Molecules in Circulation

Plasma levels osteoactivin, Syndecan-4, OPG and SPARC were assessed using a customized human premixed multi-analyte bone panel kit for Luminex assay (R&D systems, Minneapolis, MN, USA. Cat# LXSAHM). Samples were thawed on ice and centrifuged for 5 min at 10,000 ×g at 4°C to remove any remaining cells or platelets. The level of the various molecules associated with bone remodeling was measured. Plasma was diluted 2× with the sample diluent and the assay was performed following the manufacturer’s kit instructions. The assay was run on the Bio-Plex 200 system (Bio-Rad, CA, USA) and the Bio-Plex manager software was used to quantify the concentration of each analyte. The confidence level between the expected and observed concentration levels for each analyte was between 95-105% as assessed by the system.

### Statistical Analysis

The clinical data and plasma level of the different molecules in this study were statistically analyzed after categorizing the study population into the following 4 groups: ND-Nob, ND-ob, T2D-Nob and T2D-ob. One-way ANOVA was used to perform the analysis followed by a post-hoc Tukey test (Graphpad Prism Software, version 9.0). Data is reported as mean ± standard error of mean (SEM). Statistical assessments were considered statistically significant at p < 0.05. Spearman’s correlation coefficient was used to determine the association between METRNL, irisin and the osteogenic factors under study. Analyses were performed using the GraphPAD Prism Software, version 9.0.

## Results

### Characteristics of the Study Population

Selected characteristics of our sample population was studied by stratifying the population into 4 groups (ND-Nob, ND-ob, T2D-Nob and T2D-ob). The biochemical and physical characteristics such as Age, BMI, waist/hip ratio, total cholesterol (TC), high-density lipoproteins (HDL), low-density lipoprotein (LDL), triglycerides (TG), fasting blood glucose (FBG), hemoglobin A1c (HbA1c) and insulin showed statistical significance between means across all the groups. Specifically, the post-hoc analysis revealed that there was a statistically significant difference in age between the ND and T2D groups. There was no significant difference in BMI between the ND-ob and T2D-ob groups. The waist-hip ratio, HDL, TG, FBG and HbA1c showed statistically significant difference with obesity and T2D. A significant difference in insulin level was observed between the ND-Nob compared to ND-ob and T2D-ob groups (**Table 1**). Circulating levels of irisin and METRNL were significantly higher in T2D and obesity, as shown in our previous report (7).

**Table 1:** Physical and biochemical characteristics of the population characterized into 4 groups: non-diabetic non obese (ND-Nob), non-diabetic obese (ND-ob), type 2 diabetes non obese (T2D-Nob) and type 2 diabetes obese (T2D-ob).

Phenotype	ND (n = 124)		T2D (n = 104)		ANOVA	Tukey Multiple comparisons					
	Nob	ob	Nob	ob		ND-Nob			ND-ob		T2D-Nob
	(n = 73)	(n = 51)	(n = 38)	(n = 66)	Overall *p*	ND-ob	T2D-Nob	T2D-ob	T2D-Nob	T2D-ob	T2D-ob
**Age (years)**	40.5 ± 1.40	43.64 ± 1.77	51.24 ± 1.60	52.91 ± 1.11	**<0.0001**	**0.0237**	**<0.0001**	**<0.0001**	**<0.0001**	**<0.0001**	0.9159
**^†^BMI (kg/m2)**	25.31 ± 0.35	34.04 ± 0.45	26.89 ± 0.37	34.34 ± 0.30	**<0.0001**	**<0.0001**	**<0.0001**	**<0.0001**	**<0.0001**	0.7466	**<0.0001**
**Waist/Hip ratio**	0.83 ± 0.02	0.89 ± 0.01	0.92 ± 0.02	0.98 ± 0.03	**<0.0001**	ns	ns	**<0.0001**	ns	**<0.0001**	**<0.0001**
**^‡^TC (mmol/L)**	5.10 ± 0.11	5.09 ± 0.14	4.84 ± 0.27	4.93 ± 0.14	**0.0296**	0.8574	0.2524	0.3065	0.0857	0.0866	0.9719
**^§^HDL (mmol/L)**	1.39 ± 0.05	1.34 ± 0.05	1.23 ± 0.10	1.15 ± 0.05	**<0.0001**	**0.0004**	**0.0351**	**<0.0001**	0.9889	0.8067	0.742
**LDL (mmol/L)**	3.19 ± 0.1	3.21 ± 0.13	3.02 ± 0.21	3.0 ± 0.13	**0.0137**	0.6076	0.3803	0.2613	0.0709	**0.0245**	0.9993
**^§§^TGL (mmol/L)**	1.12 ± 0.12	1.214 ± 0.10	1.50 ± 0.18	1.71 ± 0.15	**<0.0001**	**0.0005**	**0.0139**	**<0.0001**	ns	0.0902	0.2734
**^††^FBG (mmol/L)**	5.23 ± 0.15	5.45 ± 0.13	7.21 ± 0.38	8.70 ± 0.41	**<0.0001**	0.7591	**<0.0001**	**<0.0001**	**<0.0001**	**<0.0001**	**<0.0001**
**^‡‡^HbA1c (DCCT%)**	5.56 ± 0.09	5.57 ± 0.08	6.66 ± 0.21	8.23 ± 0.22	**<0.0001**	0.5203	**<0.0001**	**<0.0001**	**<0.0001**	**<0.0001**	**<0.0001**
**Insulin**	8.96 ± 0.79	9.58 ± 0.91	16.80 ± 2.16	14.13 ± 1.37	**<0.0001**	**0.0001**	0.4693	**0.0016**	0.2488	0.9732	0.4678

Data are presented as mean ± SEM. One-way ANOVA test was used to compare various clinical and biochemical parameters (n=228). ^†^BMI (body mass index); ^‡^TC (total cholesterol); ^§^HDL (high-density lipoprotein); ^¶^LDL (low-density lipoprotein); ^§§^TGL (triglycerides); ^††^FBG (fasting blood glucose); ^‡‡^HbA1c (hemoglobin A1c). Tukey post-hoc analysis was performed to compare the groups. ns = >0.9999.

Bold values indicate statistically significant results.

### Expression of Osteogenic Molecules in Circulation

**Figure 1:**
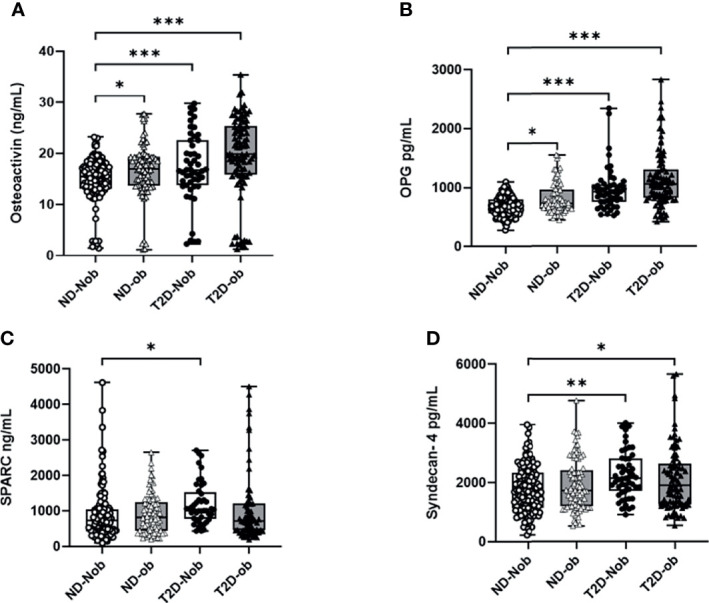
Plasma levels of osteogenic factors Comparing ND to T2D individuals classified on the basis of obesity. **(A)** Osteoactivin, **(B)** Osteoprotegerin (OPG), **(C)** Osteonectin (SPARC), **(D)** Syndecan-4. Plasma levels of various osteogenic factors was determined using a multiplex bone panel. Diabetes was defined by fasting plasma glucose ≥ 126mg/L (7 mmol/L). Obesity was defined based on BMI, where participants with BMI > 30 kg/m2 were considered ob and those with BMI between 20 and 30 kg/m2 were considered Nob. Statistical assessment was considered statistically significant at *p < 0.05, **p ≤ 0.01, ***p < 0.001.

The plasma level of osteoactivin, OPG, SPARC and Syndecan-4 expressed as mean ± SEM are presented in **Table 2**. The One-way ANOVA analysis revealed that there is a statistically significant difference in the levels of the osteogenic molecules studied across all the groups. Significantly higher level of osteoactivin was observed in people with T2D (Nob and ob) as compared to the ND people of both groups (**Figure 1A**). A slight increase was observed in osteoactivin level between the T2D-Nob and T2D-ob groups that did not reach significance. Similarly, a significant increase was observed in the level of OPG with T2D and obesity (**Figure 1B**). Interestingly the level of OPG in the ND-ob group was close to that of the T2D-Nob and there was not much difference within the T2D groups. Statistical analysis of SPARC in circulation showed a significant increase between the ND-Nob and T2D-Nob groups (**Figure 1C**). Finally, Syndecan-4 level was significantly increased with T2D (**Figure 1D**).

**Table 2:** Plasma level of molecules involved in bone remodeling in the population characterized into 4 groups: non-diabetic non obese (ND-Nob), non-diabetic obese (ND-ob), type 2 diabetes non obese (T2D-Nob) and type 2 diabetes obese (T2D-ob).

Osteogenic Factors	ND (n = 124)		T2D (n = 104)		Anova	Tukey Multiple comparisons					
	Nob	ob	Nob	ob		ND-Nob			ND-ob		T2D-Nob
	(n = 73)	(n = 51)	(n = 38)	(n = 66)	Overall *p*	ND-ob	T2D-Nob	T2D-ob	T2D-Nob	T2D-ob	T2D-ob
**Osteoactivin (ng/ml)**	16.036 ± 0.358	17.363 ± 0.499	19.54 ± 1.067	21.210 ± 0.691	**<0.0001**	**0.0472**	**<0.0001**	**<0.0001**	**0.0127**	**<0.0001**	0.2754
**OPG (pg/ml)**	696.90 ± 20.57	768.82 ± 29.70	912.80 ± 45.88	1034.330 ± 44.27	**<0.0001**	**0.0362**	**<0.0001**	**<0.0001**	0.1905	**<0.0001**	0.2966
**SPARC (ng/ml)**	795.04 ± 71.33	834.00 ± 64.65	1331.61 ± 74.49	957.920 ± 133.71	**0.0385**	0.9734	**0.0432**	0.3195	0.1433	0.6272	0.7342
**Syndecan-4 (pg/ml)**	1680.65 ± 79.69	1842.31 ± 02.01	2256.03 ± 43.08	1972.03 ± 129.43	**0.0043**	0.6909	**0.0092**	**0.0398**	0.1492	0.4621	0.8353

Data are presented as mean ± SEM. One-way ANOVA test was used to compare the level of various osteogenic molecules across different groups. Tukey post-hoc analysis was performed to compare the groups.

Bold values indicate statistically significant results.

### Correlation Analysis

**Figure 2:**
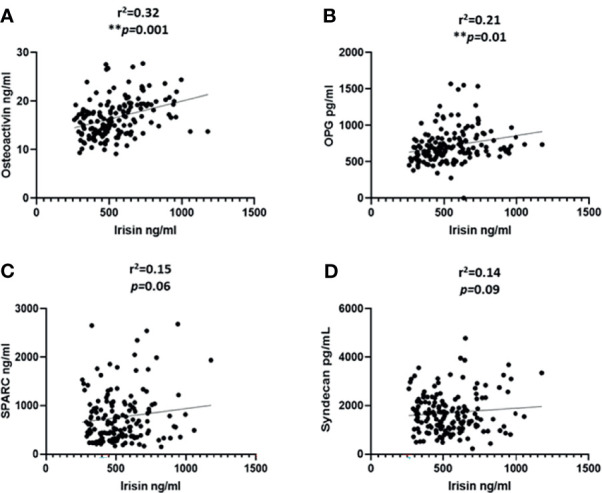
Correlation analysis between irisin and various molecules associated with bone remodeling. **(A)** Osteoactivin, **(B)** Osteoprotegerin (OPG), **(C)** Osteonectin (SPARC), **(D)** Syndecan-4 levels in plasma. Performed on the non-diabetic individuals age adjusted. Irisin level in plasma was determined using enzyme linked immunosorbent assay (ELISA), a customized multiplex bone panel was used to assess the level of various osteogenic molecules. Diabetes was defined by fasting plasma glucose ≥ 126mg/L (7 mmol/L). Spearman correlation coefficient was used to determine the association of irisin with the different osteogenic factors. Statistical assessment was 2-sided and considered statistically significant at **p ≤ 0.01.

**Figure 3:**
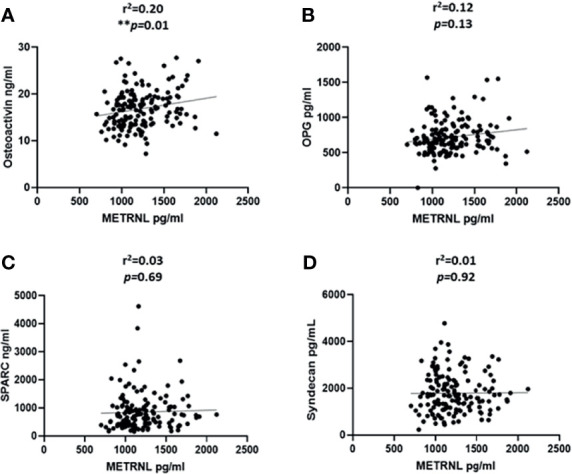
Correlation analysis between METRNL and various molecules associated with bone remodeling. **(A)** Osteoactivin, **(B)** Osteoprotegerin (OPG), **(C)** Osteonectin (SPARC), **(D)** Syndecan-4 levels in plasma. Performed on the non-diabetic individuals age adjusted. METRNL level in plasma was determined using enzyme linked immunosorbent assay (ELISA), the circulating level of various osteogenic molecules were determined using a customized multiplex bone panel. Diabetes was defined by fasting plasma glucose ≥ 126mg/L (7 mmol/L). Spearman correlation coefficient was used to determine the association of METRNL with the different osteogenic factors. Statistical assessment was 2-sided and considered statistically significant at **p ≤ 0.01.

We categorized the population based on their diabetes status; the correlation analysis was performed only on the non-diabetic population. This was done to exclude the effect of T2D related drugs. The gender distribution was equal and the analysis was performed adjusted for age. We observed a significant correlation between the level of irisin with osteoactivin (r^2^ = 0.32 and P < 0.0001) (**Figure 2A**). Irisin also showed a significant correlation with OPG (r^2^ = 0.21 and P = 0.01) (**Figure 2B**). No association was observed between the levels of irisin with SPARC (**Figure 2C**) and Syndecan-4 (**Figure 2D**). The correlation analysis of the level of METRNL with the various osteogenic molecules under study, showed a significant association between METRNL and osteoactivin (r^2^ =0.2 and P = 0.01) (**Figure 3A**). However no association was seen between METRNL with OPG, SPARC and Syndecan-4 (**Figure 3B–D**).

## Discussion

In this study plasma levels of osteoactivin and OPG were shown to be increased with obesity and T2D concomitant with increased levels of irisin and METRNL. Additionally, the data showed increased plasma levels of SPARC and Syndecan-4 only in the T2D group. For the first time, within the non-diabetic population, a positive correlation was seen between osteoactivin and irisin as well as with METRNL (the two main adipomyokines of our interest). A positive correlation was also observed between irisin and OPG. These associations are particularly interesting as these osteogenic factors are secreted by osteoblasts and contribute to regulating bone cell differentiation (24).

Beyond its function in physically and mechanically supporting the body, the musculoskeletal system has been shown to secrete various hormones that are involved in regulating metabolic and inflammatory pathways. One of these molecules that plays an important role in osteoblast differentiation and function is osteoactivin, also known as glycoprotein non-metastatic melanomal protein B (GPNMB). It is a type 1 transmembrane glycoprotein that is found on the cell surface or stored in endosomes/lysosomes. It can be cleaved to generate a soluble isoform (25, 26). Originally discovered in melanoma cell lines (27), osteoblasts (28) and dendritic cells (29), the expression of osteoactivin was also detected in osteoclasts, skin melanocytes, retinal pigment epithelial cells, hepatocytes and leukocytes (30). The wide array of osteoactivin expression explains its involvement in numerous physiological and pathological cellular processes. Osteoactivin is a positive regulator of osteoblast differentiation and has been recently identified as a regulator of lipogenesis in WAT and was also found to exacerbate diet induced obesity and insulin resistance (31). Microarray and transcriptomic analysis done on animal models revealed that osteoactivin is one of the highly upregulated genes in fat tissues (32–34). Osteoactivin is also suggested to be a regulator of inflammation in the adipose tissue favoring tissue protection and repair (35–38), which was corroborated by an increased expression in anti-inflammatory M2 macrophages in comparison to the pro-inflammatory M1 macrophages (39). Several studies have reported increased levels of osteoactivin in circulation associated with various metabolic disorders (40–42). The data from this study agrees with these studies and demonstrates an overexpression of osteoactivin in people with obesity and T2D. This could implicate its possible involvement in combatting the adverse effects or it could also be an outcome of complications associated with obesity and T2D. Especially that osteoactivin is expressed and secreted by various tissues therefore it may play a role locally within the tissue or systemically through circulation.

OPG is a member of the tumor necrosis factor (TNF) receptor superfamily, originally characterized by its ability to suppress osteoclast formation (43, 44). OPG is expressed by different cells, including osteoblasts, osteoclast precursors, mature osteoclasts, dendritic cells, B and T cells, fibroblasts, intestinal epithelial cells, vascular endothelial cells, and some cancer cells (43, 45). In a study involving Chinese postmenopausal women with either prediabetes or T2D, serum OPG was significantly associated with HOMA-IR and a significant increase in OPG levels was associated with impaired glucose regulation (46). Numerous clinical studies have also reported an association between increased serum OPG and coronary artery disease (CAD) and/or cardiovascular complications and mortality (47). Nonetheless, the reported increase in serum OPG levels is an insufficient compensatory or defense response to prevent vascular endothelial dysfunction and the progression of atherosclerosis (48). The data obtained from this study is in accordance with previous reports, that showed increased level of OPG in people with Obesity and T2D (43). It is however, still to be elucidated whether this overexpression of OPG is protective or detrimental in the development of complications linked to Obesity and T2D.

Osteonectin also known as secreted protein acidic and rich in cysteine (SPARC), was originally identified as a bone specific protein (49), it is an abundantly expressed glycoprotein that plays an important role in bone mineralization, cell matrix interactions and collagen binding (50). Elevated plasma level of SPARC have shown to be strongly associated with insulin resistance, dyslipidemia and inflammation (51, 52). The data from this study agrees with the reported finding of increased level of SPARC in plasma with T2D. Similarly, Syndecan-4, which is a proteoglycan adhesion receptor, considered a coreceptor to integrins, was seen to be increased with T2D. This agrees with a previous report showing that the expression of this molecule, in circulation, is increased with T2D (53). This protein is also shown to be involved in bone formation and it has been reported that loss of Syndecan-4 impairs adult fracture healing (54).

In summary, the results obtained from the current study concurs with previous reports that show a dysregulation in the expression level of osteoactivin, OPG, SPARC and Syndecan-4 in circulation with metabolic disease state. Considering that these molecules are all involved in inflammation and bone formation, it is suggested that this dysregulation maybe the result of the low-grade inflammation, which could lead to bone related complications associated with these conditions.

Both Irisin and METRNL are primarily secreted by the skeletal muscles. It has been reported that irisin plays an essential role in the interaction between muscle tissue and bone metabolism, suggesting that there is a strong bone-muscle interaction (9, 55–57). Recently there has been a stronger link between irisin and bone metabolism through the identification of an integrin αV/β5 that acts as the irisin receptor on osteocytes (58). The authors of this study also showed that irisin treatment led to increased sclerostin level in osteocyte-like cells. This is in contrast with what was previously reported by Colaianni et al. where irisin level was negatively associated with sclerostin levels (59). These conflicting data were thought to stem from a fundamental paradigm difference in the function of irisin, whereby it might be similar to parathyroid hormone (PTH) that exerts both anabolic and catabolic effects on the skeleton. It was postulated that the rise in irisin levels for an extended period of time can lead to increased bone catabolism, while its knockout can lead to bone formation (60). Nevertheless, irisin was shown to play an important role in increasing bone formation *via* regulating osteoblast-led bone formation (57, 59, 61). Interestingly, the data from the current study highlights a strong association between irisin and two factors involved in bone remodeling namely, osteoactivin and OPG. The interaction between irisin and osteoactivin may be through their role in the process of osteoblast differentiation as both exert their function by binding to integrin receptors (58, 62). Furthermore, it has been reported that treatment with irisin induces osteoblast differentiation, which in turn resulted in an increase in OPG levels (16, 63). The association observed through the current study between the levels of irisin and OPG agrees with these findings. Similarly, in a study investigating the genes involved in osteoblast differentiation, it was shown that METRNL has a unique expression pattern in bone and may inhibit bone cell differentiation (22). The interaction between MERNL and osteoactivin further highlights the importance of these molecules in the muscle and bone crosstalk. Adipomyokines, are also known to play a role in maintaining systemic low-grade inflammation during the progression of metabolic diseases. Considering the osteogenic molecules also display a parallel dysregulation in metabolic disease conditions, the association observed between these molecules sheds light on the possible interaction of these molecules during the development and progression of metabolic diseases.

One of the main limitations of the current study is its cross-sectional study design. This makes it difficult to decipher the actual interaction between both irisin and METRNL with osteoactivin and OPG. As a result, more functional analysis to better understand the interaction between these markers and establish their role in metabolic diseases need to be performed. Furthermore, this study emphasized the role of some of the molecules involved in bone remodeling. Therefore, it is of interest to expand the study and investigate the expression of other important bone related molecules as well as to investigate the effect of this dysregulation on bone mineral density and bone mineral content to get a better understanding on the relationship between bone and muscle crosstalk in obesity and T2D.

In conclusion, our findings suggest that there is a dysregulation in the expression of various markers in the musculoskeletal system mainly irisin, METRNL, osteoactivin and OPG, which may result in the development of various complications associated with obesity and T2D. We also suggest that there is a possible crosstalk between adipomyokines and osteogenic factors that may play a possible role in the development of bone and muscle related complications in association to obesity and T2D. Further studies are necessary to understand the mechanistic significance of their possible interplay.

## Data Availability Statement

The data supporting the findings of this study are available within the article. Raw data that support the findings of this study are available through the corresponding authors upon reasonable request.

## Ethics Statement

The study was approved by the ethical review board of Dasman Diabetes Institute and has been conducted in accordance with the ethical guidelines of the Declaration of Helsinki. The patients/participants provided their written informed consent to participate in this study.

## Author Contributions

FA-M, MA-F and JA designed, helped in writing, reviewed the manuscript and supervised the experiments. IA-K and PC performed the experiments and wrote the manuscript. MJ, SA-S, HA, EA, GA-K, CD and WA-A critically reviewed the manuscript. IA-K and PC contributed equally to this work. All authors contributed to the article and approved the submitted version.

## Funding

This research was funded by Kuwait Foundation for the Advancement of Sciences (KFAS), grant number RA-2016-045.

## Conflict of Interest

The authors declare that the research was conducted in the absence of any commercial or financial relationships that could be construed as a potential conflict of interest.

## Publisher’s Note

All claims expressed in this article are solely those of the authors and do not necessarily represent those of their affiliated organizations, or those of the publisher, the editors and the reviewers. Any product that may be evaluated in this article, or claim that may be made by its manufacturer, is not guaranteed or endorsed by the publisher.
